# Research on Robotic Compliance Control for Ultrasonic Strengthening of Aviation Blade Surface

**DOI:** 10.3390/mi14040730

**Published:** 2023-03-25

**Authors:** Shanxiang Fang, Yao Du, Yong Zhang, Fanbo Meng, Marcelo H. Ang

**Affiliations:** 1 Department of Mathematics and Theory, Peng Cheng Laboratory, Shenzhen 518055, China; 2 Department of Mechanical Engineering, National University of Singapore, Singapore 117575, Singapore; 3 VIBOT/ImViA, IUT, Université Bourgogne Franche-Comté, 9 avenue Alain Savary, BP 47870, 21078 Dijon Cedex, France; 4 School of Mechano-Electronic Engineering, Xidian University, Xi’an 710071, China

**Keywords:** industry robot, compliance control, neural network fuzzy PID control, ultrasonic strengthening, aviation blade surface

## Abstract

In order to satisfy the requirement of the automatic ultrasonic strengthening of an aviation blade surface, this paper puts forward a robotic compliance control strategy of contact force for ultrasonic surface strengthening. By building the force/position control method for robotic ultrasonic surface strengthening., the compliant output of the contact force is achieved by using the robot’s end-effector (compliant force control device). Based on the control model of the end-effector obtained from experimental determination, a fuzzy neural network PID control is used to optimize the compliance control system, which improves the adjustment accuracy and tracking performance of the system. An experimental platform is built to verify the effectiveness and feasibility of the compliance control strategy for the robotic ultrasonic strengthening of an aviation blade surface. The results demonstrate that the proposed method maintains the compliant contact between the ultrasonic strengthening tool and the blade surface under multi-impact and vibration conditions.

## 1. Introduction

The surface strengthening of a titanium alloy aviation blade holds significant importance in improving an aircraft’s flight safety, as well as the service life and work efficiency of its engine [1,2,3]. Ultrasonic surface strengthening (USS) is an innovative surface treatment technology, which has the ability to ameliorate the hardness, surface roughness, and wear resistance of the processed workpiece’s surface [4]. To improve the automation level of ultrasonic strengthening and the surface quality of the blade surface, it is necessary to load the strengthening device on the industrial robot to complete the process automatically. In the ultrasonic strengthening process, the contact force between the strengthening tool and the workpiece surface is vital for forming a residual stress layer [5]. The constant contact force not only maintains the oscillation stability of the strengthening tool but also induces elastic–plastic deformation of the workpiece material, making it a crucial factor [6,7]. Therefore, compliance control is required during the process of robotic ultrasonic surface strengthening to keep a constant contact force.

The compliance control strategy encompasses a passive compliance mechanism, force feedback control, and impedance control, any one of which, when applied independently, cannot effectively solve the control problem of contact force in robotic machining [8,9,10,11,12,13]. As a robotic control study develops into an advanced stage, intelligent control methods are used to tackle the control problem of a complex system.

Neural networks are capable of self-organization, self-learning, nonlinear approximation, distributed storage, and the parallel processing of information [14,15,16,17]. They can simulate human thinking and reflect certain characteristics of human brain function. Neural networks are widely used in the high-precision control of intelligent industrial robots [18,19,20]. For instance, Shah et al. [21] designed a four-layer neural network to solve the path, time, and power optimization problems of robots, and the cognitive tasks involved included learning, adaptation, induction, and optimization. Zhang et al. [22] proposed a dynamic compensatory strategy for industrial robot manipulators. The parameters of the uncertain dynamics model were estimated by the RBF neural network, while a variable structure controller was employed to make up the approximation errors of the neural network. With the help of the robust H∞ controller, the effect of external disturbances will be attenuated, and robust tracking performance will be achieved. As for the time-varying characteristics of the transfer function of industrial robots in high-speed motion, Wang et al. [23] derived a relationship model between the motion state parameters of manipulators and PID parameter values through neural networks, and constructed a neural network PID controller to improve the accuracy of industrial robot tracking algorithms. The dynamic control system of industrial robots has multi-input, multi-output, nonlinear, strong coupling, and time-varying characteristics. Truong et al. [24] used a neural network algorithm to optimize the dynamic part of industrial robots, thereby significantly improving the robot’s force track and anti-interference abilities. Additionally, the final surface machining quality of a workpiece is affected by various factors, including robot machining contact force, feed rate, machining repetition rate, and machining path planning, among which contact force is essential [25,26,27]. Thus, it is necessary to develop different compliance force control methods for different working conditions, analyze the machining process, and summarize the knowledge of the combination of process parameters to lay a theoretical foundation for the automatic strengthening of an aviation blade surface.

In current complex production and processing systems, industrial robots need to have strong comprehensive information processing capabilities [28,29]. To meet this challenge, the intelligent compliance control system is widely used in industrial fields due to its self-adaptation, organization, coordination, optimization, and variable structure [30,31,32]. However, for specific operation tasks, discovering how to combine the control method with a specific application is a very complex problem. Therefore, the force control during ultrasonic surface strengthening is analyzed in this work, the goal of which is to use compliance control to maintain constant contact force under multi-impact and vibration conditions. The existing compliance control method for grinding robots can provide a relevant technical reference. By establishing the dynamic relationship between machining force and robotic end-position deviation, the robot can maintain the appropriate output force in a constraint environment.

This paper proposes a robotic compliance control method of contact force for ultrasonic surface strengthening. With the combined control method of a fuzzy PID controller and the RBF neural network, the compliance control of output force is realized based on the end-effector. This approach keeps the contact force between the ultrasonic tool and the blade surface constant during ultrasonic strengthening.

## 2. Force/Position Control Method for Robotic Ultrasonic Surface Strengthening

It is necessary to keep constant normal contact pressure during the ultrasonic strengthening of the aviation blade surface. This means that the motion path of the robot end and the output force in the *Z*-axis of the TCP coordinate system shall be controlled simultaneously in the strengthening process. To solve this problem, it is necessary to design the force/position control method for ultrasonic surface strengthening. However, due to the large rigidity of industrial robots, aviation blade workpieces, and the working environment, the control model must be highly accurate to obtain the expected end position and output contact force [33]. Indeed, it is difficult to realize in practice. In addition, the coupling of the position control and the force control also makes the control system more complex.

**Figure 1:**
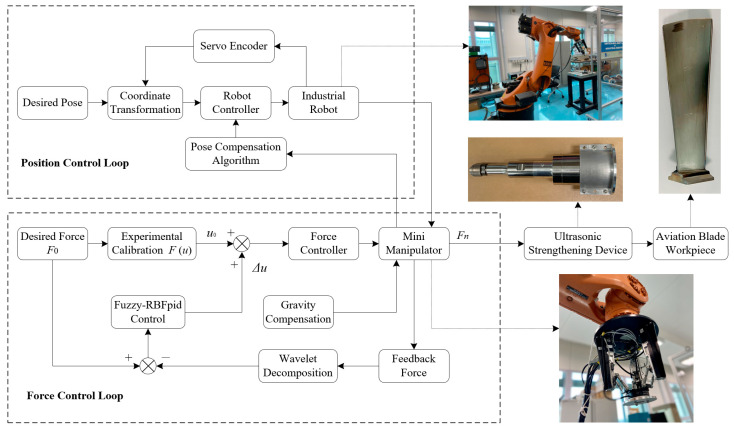
Force/position control schematic of robotic ultrasonic surface strengthening.

The force control method, here, applies an end-effector (mini-manipulator), as shown in Figure 1. In the process of ultrasonic surface strengthening, the force control and position control are decoupled at the end of the strengthening tool, on which neither will constitute constraint. The position control is realized by the KUKA KR60 robot, while the force control is realized by the end-effector. The coupling control problem between position and force in the system can be solved. Thus, it is easier to realize force/position control of the robotic ultrasonic strengthening system due to such a scattered control method.

In the force control loop, the expected contact force *F*_0_ is transformed into the control variable *u*_0_; the force control signal is output to the end-effector by the force controller, with output force *F_n_*. In consideration of the gravity compensation of the transducer, the contact force of the ultrasonic strengthening tool exerted on the blade surface is *F_c_*. A six-axis force/torque sensor (SRI M3704B) is mounted at the wrist of the end-effector, and the measured feedback force is *F_s_*, which can indicate the contact force between the strengthening tool and the workpiece surface in a control cycle. The feedback force *F_s_* is compared with the expected contact force *F*_0_ to adjust the control quantity by the compliance controller until the control deviation Δ*u* is 0, and the contact force for strengthening *F_c_* is the expected force *F*_0_. The gravity compensation compensates for the component force of the ultrasonic strengthening tool’s gravity in the contact direction, which has a variation of a sinusoidal function with the end posture adjustment of the ultrasonic strengthening robot.

## 3. Transfer Function Model of the End-Effector

The end-effector system consists of several components such as motors, lead screws, dampers, and parallel mechanisms. Referring to the control principle of a servo motor and the motion principle of a ball screw, by setting the input voltage signal, motors output the torque, and the parallel mechanism is driven by lead screws to output the Z-direction pressure. There are many nonlinear factors in the control model of the device that stop it from being quantified accurately [12]. In order to control the end-effector system, it can be treated as a “black box”, and its control model can be determined through the parameter identification method.

**Figure 2:**
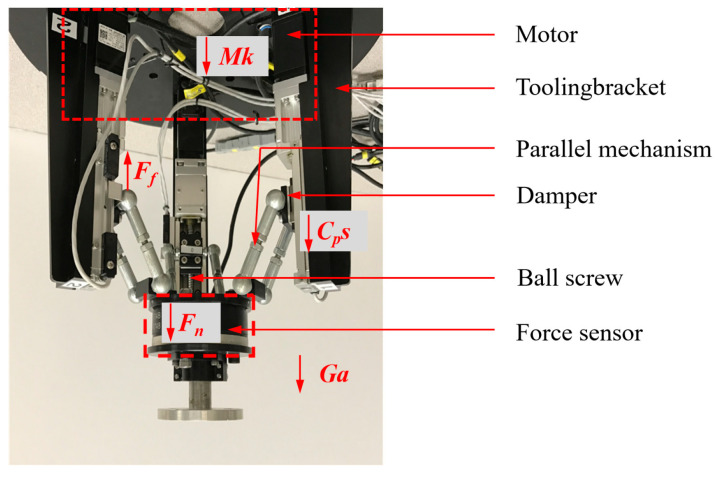
Force analysis of the end-effector.

Figure 2 shows the force analysis in the process of the end-effector outputting force. According to Newton’s second law, the force balance equation can be obtained as Equation (1).

(1) $$Mk-F_{f}=G\frac{d^{2}y}{dt^{2}}+C_{p}\frac{dy}{dt}+F_{n}$$

where *M* is the output torque of the motor, *k* is the proportionality coefficient, *F_f_* is the friction force, *G* is the total mass of the moving parts of the device, *C_p_* is the frictional damping coefficient, *y* is the displacement of the damper, and *F_n_* is the output force of the end-effector.

The main moving component is the screw guide, and the influence of low friction can be ignored. The Laplace transform for Equation (1) can be expressed as Equation (2).

(2) $$F_{n}\left(s\right)=K_{e}Y\left(s\right)$$

When the blade is strengthened, the reaction force of *F_n_* makes the end-effector passively generate displacement *y*, which can be expressed as Equation (3).

(3) $$M\left(s\right)k=Gs^{2}Y\left(s\right)+C_{p}sY\left(s\right)+F_{n}\left(s\right)$$

where *K_e_* is the equivalent stiffness coefficient.

Equation (4) can be calculated by combining Equations (2) and (3).

(4) $$\frac{F_{n}\left(s\right)}{M\left(s\right)}=\frac{K_{e}k}{Gs^{2}+C_{p}s+K_{e}}$$

For a motor, the simplified transfer function, ignoring its electromagnetic inertia, can be expressed as:

(5) $$G\left(s\right)=\frac{M\left(s\right)}{U\left(s\right)}=\frac{K_{m}}{T_{S}s+1}$$

where *K_m_* is the forward gain and *T_s_* is the inertia time coefficient of the motor transfer function.

**Figure 3:**
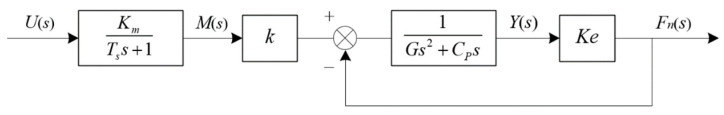
The transfer function block diagram of the end-effector system.

According to Equations (2)–(5), the transfer function block diagram of the end-effector system can be obtained, as shown in Figure 3.

The transfer function of the end-effector system can be calculated by combining Equations (4) and (5). Equation (6) is the relationship model between input control quantity and output contact force. From this equation, the control model of the end-effector proves to be a third-order system.

(6) $$G\left(s\right)=\frac{K_{e}K_{m}k}{\left(Gs^{2}+C_{p}s+K_{e}\right)\left(T_{s}s+1\right)}$$

The time domain measurement method is used to measure the model. To improve the accuracy of the measurement model, according to Equation (7), the n-order inertial link is used for fitting.

(7) $$G\left(s\right)=\frac{K}{{\left(T_{s}s+1\right)}^{n}}e^{-\tau s}$$

**Figure 4:**
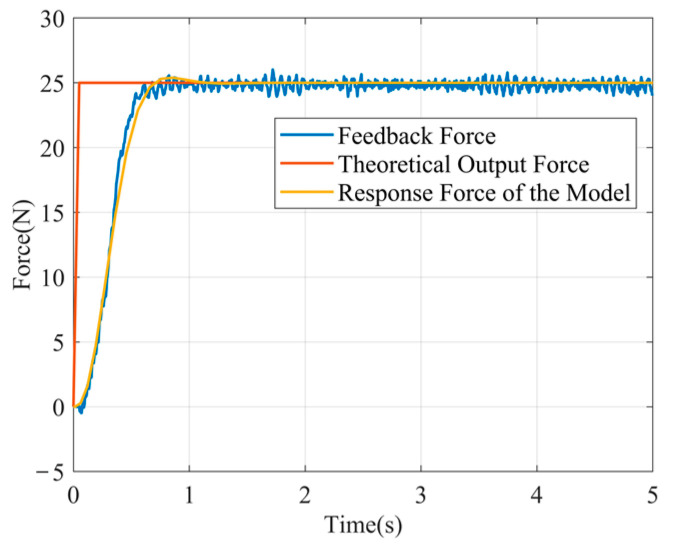
Response validation of the end-effector’s measurement model.

The blue curve in Figure 4 is the actual output force (feedback force). According to the curve data, the parameters of the end-effector control model are identified as Equation (8). By simplification, the experimental measurement model can be obtained as Equation (9).

(8) $$G\left(s\right)=\frac{K}{{\left(Ts+1\right)}^{n}}e^{-\tau s}=\frac{11.374}{{\left(0.053s+1\right)}^{5}}e^{-0.065s}$$

(9) $$G\left(s\right)=\frac{11.38}{\left(0.03s^{2}+0.27s+1\right)\left(0.07s+1\right)}=\frac{11.38}{0.0021s^{3}+0.049s^{2}+0.34s+1}$$

The yellow curve in Figure 4 is the output force response curve of the measurement model, which is generally close to the curve of the actual output force. It is shown that the output force of the end-effector system does not overshoot after applying the step signal, which proves that the system model has good stability. Therefore, the measurement model can restore the actual model of the end-effector and can be used as an approximate control model.

## 4. Fuzzy Neural Network PID Control of Output Contact Force

**Figure 5:**
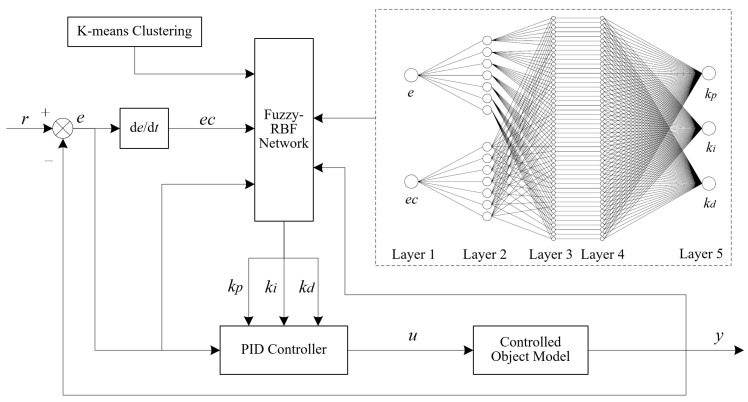
Structure diagram of the fuzzy neural network PID control system.

A fuzzy neural network control utilizes the advantages of a neural network and a fuzzy system so that the fuzzy system has the ability to self-organize and self-learn, which can effectively improve the accuracy, robustness, and self-adaptation of the system [34,35]. To solve the regulation effect’s deficiency when the conventional PID control is applied to the time-varying system, the fuzzy RBF neural network is used to self-tune the three parameters *k_p_*, *k_i_*, and *k_d_*. Figure 5 shows the structure diagram of the control system.

According to the basic principle of the control method, the system deviation *e* and its variation rate *ec* are input into the fuzzy RBF neural network as input variables and will be checked online in the control process. Then, self-tuning is conducted for the three parameters of PID based on the mapping relationship between the input variable and the output variable in the control network to select the optimal parameter of PID, so that the traditional PID controller will have self-adaptability. The initial parameters of the fuzzy RBF neural network are determined by K-means clustering to improve the training speed and approximation effect of the control network.

### 4.1. Design of the Fuzzy-RBF-PID Controller

According to the design requirements of the Fuzzy-RBF-PID controller for contact force control, the control network is designed into a five-layer structure with two inputs and three outputs. The system deviation *e* and its variation rate *ec* correspond to the two input nodes, and the PID parameters (*k_p_*, *k_i_*, *k_d_*) correspond to three output nodes. The control network structure is shown in Figure 5.

The propagation of signals in the fuzzy RBF neural network and the functions of each layer are explained as follows.

Layer 1: The two nodes of this layer are connected with the input deviation *e* and the deviation change rate *ec*, and the function is to pass the transformed *e* and *ec* to the next layer. The input and output relationship of the nodes in this layer is:

(10) $$f_{1}=\left(i\right)=X=\left[x_{1},x_{2}\right],i=1,2$$

Layer 2: The function of this layer is to transform the deterministic input quantity into a fuzzy vector and then convert the input variable into the corresponding membership degree through the membership function of the defined fuzzy subset. The membership function is a Gaussian function, and the membership of each input component is:

(11) $$f_{2}\left(i,j\right)=\exp\left(-\frac{{\left(f_{1}\left(i\right)-c_{ij}\right)}^{2}}{{\left(b_{ij}\right)}^{2}}\right),i=1,2,j=1,2,\cdots ,L$$

where *b_ij_* and *c_ij_* are the mean and standard deviation of the membership function corresponding to the *i*-th input variable and the *j*-th fuzzy set, respectively, and the number of fuzzy segmentations *L* for both input variables is set to 7.

Layer 3: In this layer, each node represents a fuzzy rule, and the fuzzy rules are matched by connecting the fuzzy layers. By combining each fuzzy node, the corresponding activation threshold can be obtained in order to complete the fuzzy inference between the nodes. The output of each node is expressed as the product of all its input signals. It can be expressed as Equation (12).

(12) $$f_{3}\left(j\right)=\prod_{j=1}^{N}f_{2}\left(i,j\right),N=\prod_{i=1}^{n}N_{i}$$

where *N_i_* is the number of the *i*-th input membership function of the input layer and *N* is the total number of nodes in the fuzzy rule layer, *N* = 49.

Layer 4: This layer normalizes the fitness of each fuzzy rule in the network.

(13) $$f_{4}\left(j\right)=\frac{f_{3}\left(j\right)}{\sum_{i=1}^{N}f_{3}\left(i\right)},j=1,2,\cdots ,N$$

Layer 5: This layer consists of three nodes, and its main function is to convert the fuzzy output quantity into a clear quantity, which, in this case, is the three parameters of PID.

(14) $$f_{5}\left(l\right)=f_{4}\cdot W=\sum_{j=1}^{N}f_{4}\left(j\right)\cdot w\left(l,j\right)$$

where ***W*** is the connection weight matrix between the output layer node and each node in the rule layer, and *f*_5_ (*l*) is the adjustment value of the three parameters *k_p_*, *k_i_*, and *k_d_*, *l* = 1, 2, 3.

The output of the fuzzy neural network PID controller is:

(15) $$\Delta u=k_{p}e\left(k\right)+k_{i}\left[e\left(k\right)-e\left(k\right)-1\right]+\alpha_{d}\left[e\left(k\right)-2e\left(k-1\right)+e\left(k-2\right)\right]$$

An incremental PID control algorithm is adopted:

(16) Δu=u(k−1)+Δu(k)

Delta (*δ*) learning rule is used to modify the adjustable parameter of the control network. Using the gradient descent method, the output error of the network will decrease by gradient with the increase in training times so that the actual output value of the system can be approximated to the ideal output value in a statistical sense.

The objective function is defined as:

(17) $$E\left(k\right)=\frac{1}{2}{\left[r\left(k\right)-y\left(k\right)\right]}^{2}$$

where *r*(*k*) is the expected output and *y*(*k*) is the actual output in each iteration, and *r*(*k*)−*y*(*k*) represents the control error. The network weight is adjusted by the following learning algorithm:

(18) $$\Delta w_{j}\left(k\right)=-\eta \frac{\partial E}{\partial w_{ij}}=-\eta \cdot e\left(k\right)\cdot f_{3}\left(j\right)$$

The learning algorithm of Δ*c_ij_* and Δ*b_ij_* in the fuzzy layer is:

(19) $$\Delta c_{ij}\left(k\right)=-\eta \frac{\partial E}{\partial c_{ij}}=-\eta \cdot e\left(k\right)\cdot w_{j}\left(k\right)\cdot \frac{2\left(x_{i}-c_{ij}\right)}{b_{j}^{2}},\;\Delta b_{ij}\left(k\right)=-\eta \frac{\partial E}{\partial b_{ij}}=-\eta \cdot e\left(k\right)\cdot f_{3}\left(j\right)\cdot \frac{2\left(x_{i}-c_{ij}\right)}{b_{j}^{2}}$$

Considering the momentum factor, the weight of the output layer can be expressed as:

(20) $$w_{j}\left(k\right)=w_{j}\left(k-1\right)+\Delta w_{j}\left(k\right)+\alpha \left[w_{j}\left(k-1\right)-w_{j}\left(k-2\right)\right]$$

where *k* is the iterative step of learning algorithms, *η* is the learning rate, *η* ∈ (0, 1), and *α* is the momentum factor, *α* ∈ (0, 1).

### 4.2. Simulation of Fuzzy Neural Network PID Control for Contact Force

The regulation and tracking performance of the control algorithm is very important for nonlinear systems with uncertain dynamics and external disturbances [36,37]. To verify the control performance of the Fuzzy-RBF-PID controller for contact force, the step response and positive sinusoidal tracking response of the output force are simulated.

**Figure 6:**
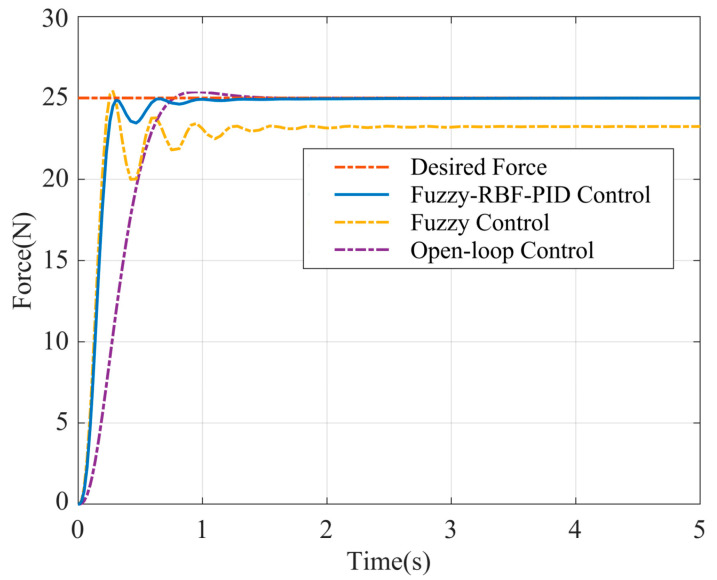
Step response curve of Fuzzy-RBF-PID control.

When *t* = 0, the input signal *F*_0_ = 25 N, and the step response curve of the system output force is obtained, as shown in Figure 6. The simulation results show that the step response time of the output force for open-loop control with the experimental measurement model is 0.76 s, and that there is a 1.9% overshoot. When the Fuzzy-RBF-PID control module is added, the step response speed of the system output force is significantly improved, and the response time is reduced to 0.31 s. The response curve quickly enters a stable state after two small-amplitude oscillations and has nearly no overshoot, thus improving both the stability precision and the response performance of the system.

**Figure 7:**
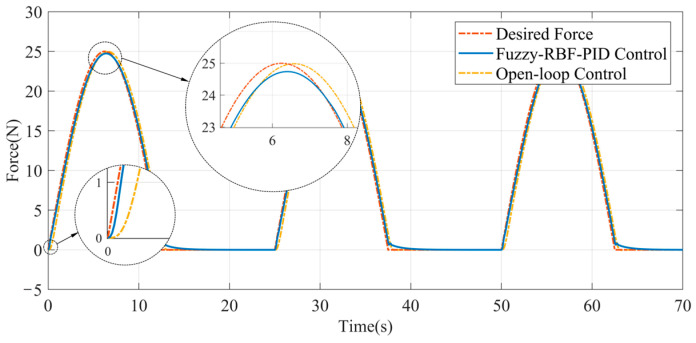
Forward sinusoidal response curve of Fuzzy-RBF-PID control.

**Figure 8:**
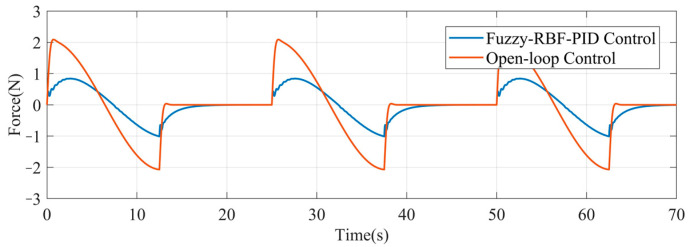
Dynamic tracking error of the output force.

Figure 7 shows the sinusoidal tracking response curve. The expected force signal is a sinusoidal signal with the amplitude of 25 N and a frequency of 0.04 Hz. Since the actual output force of the end-effector is not less than zero, the negative part of the input sinusoidal signal is filtered out. The figure shows that the system will achieve good overall tracking performance after the addition of the Fuzzy-RBF-PID control module. The response curve oscillates slightly near the starting point and then quickly enters the tracking state. In addition, the tuning delay is substantially smaller. As shown in Figure 8, the Fuzzy-RBF-PID control decreases the dynamic tracking error of the output force from ±2.1 N to ±1 N.

Therefore, the Fuzzy-RBF-PID control method can increase the adjusting speed, reduce overshoot, improve dynamic response performance, maintain a good tracking accuracy, and significantly improve the adjusting performance of the end-effector and the tracking robustness of the dynamic system.

## 5. Experiment Research on Robotic Compliance Control for Ultrasonic Strengthening of Aviation Blade Surface

**Figure 9:**
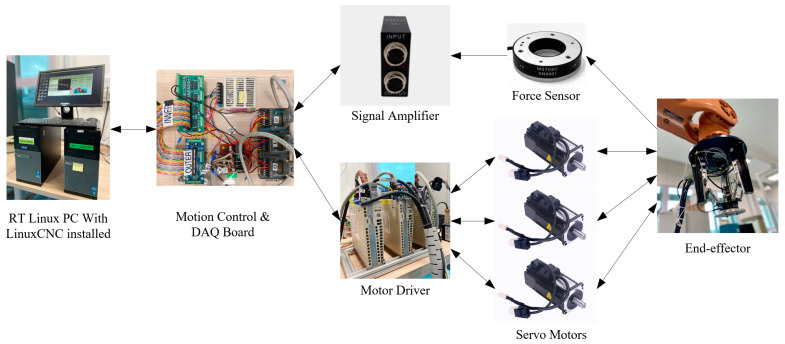
The structure of the end-effector’s hardware control platform.

The end-effector is loaded at the end of the KUKA KR60 industrial robot for experimental research. The structure of the end-effector’s hardware control platform is shown in Figure 9. The Realtime Application Interface (RTAI) is adopted and LinuxCNC is used as the control platform. The interface card, Motenc-100, is used for signal transformation and communication between the servo motor and sensor. The six-dimensional force sensor M3704B is used in the experiment, which can be used to test the force in *X*-axis, *Y*-axis and *Z*-axis directions and the torque around these three directions in the space. This sensor has a range of ±200 N in the *Z*-axis direction and a steady-state error of 0.1 N-0.2 N.

### 5.1. Experiment on the Compliance Control Algorithm of Contact Force

To evaluate the performance improvement of the fuzzy neural network PID controller on the end-effector system’s response, an experimental analysis is conducted using four control methods: open-loop control, PID control, fuzzy control, and Fuzzy-RBF-PID control. The control algorithm is implemented by using Labview, and the sampling frequency is set to 1 ms. For each control method, two groups of experiments are conducted: a step response experiment to analyze the system’s response performance and control accuracy, and a sinusoidal tracking experiment to analyze the system’s tracking robustness.

#### 5.1.1. Step Response Analysis

**Figure 10:**
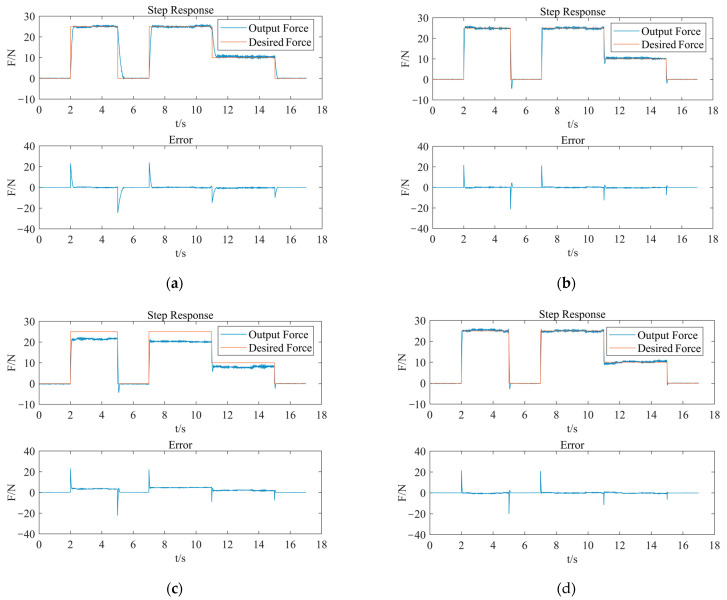
Step response analysis of contact force. (**a**) Open-loop control; (**b**) PID control; (**c**) Fuzzy control; (**d**) Fuzzy-RBF-PID control.

As shown in Figure 10, from the PID control response curve, overshoot can almost not be seen in the rising stage, but appears for a short time in the descent stage. In the 25-0 N descent stage, the overshoot lasts for about 176 ms. The response error curve shows a specific deviation in the control system, but the duration is short.

The adjusting speed of fuzzy control is slightly faster than that of PID control. In terms of adjusting deviation, when the expected output force signal is 25 N, the feedback force will be maintained at about 20 N; when the expected output force signal is set to 10 N, the feedback force will be maintained at about 8.5 N. There are two main reasons for the steady-state error. First, the fuzzy rules and control parameters, such as the quantification factor, are not set appropriately. Second, the fuzzy controller inputs deviation *e* and its variation rate *ec*, equivalent to a PD controller lacking the integration element. After fuzzification, the system’s control output is constrained within the fixed domain. Therefore, the system uses the fuzzy control, of which the output variable will inevitably have a steady-state error.

By combining fuzzy control with neural network control, a fuzzy neural network PID controller is designed. According to the step response curve of the same expected force, it can be analyzed that the use of fuzzy neural network PID control can make the adjusting speed of the output force in the descending region significantly faster. Furthermore, there is no overshoot in the rising stage of the system, but occasionally it can be observed in the descending stage (about 9%), in which it lasts for about 60 ms. These values are smaller than the PID control method. In comparison to the fuzzy control, the control deviation in the expected force maintenance stage is eliminated. Consequently, the fuzzy neural network PID controller exhibits superior control performance compared to the common PID controller and fuzzy controller. The system can be adjusted more accurately and quickly, while its steady-state accuracy can be guaranteed.

#### 5.1.2. Sinusoidal Tracking Response Analysis

**Figure 11:**
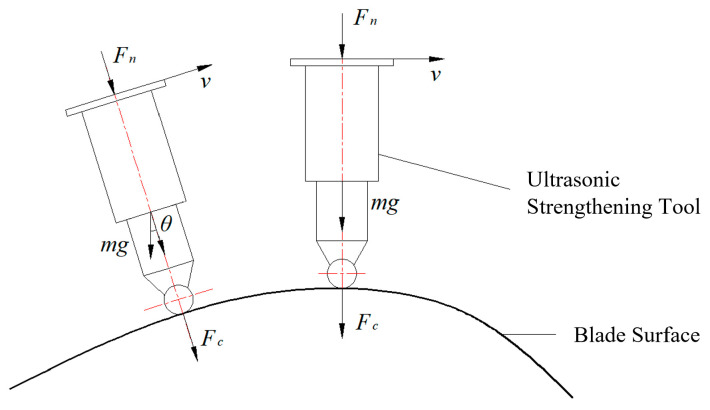
Force analysis of strengthening tool in the strengthening process.

The ultrasonic strengthening tool is mounted on the force sensor fixed to the end-effector. In the process of the robotic ultrasonic strengthening of an aviation blade surface, it is necessary to adjust the posture of the tool with the contact position in order to ensure that the contact force has normal pressure, as shown in Figure 11. Here, *F_c_* is the contact force, *F_n_* is the output force of the end-effector, *θ* is the angle between the end-effector and vertical direction, and the output force can be expressed as Equation (21).

(21) $$F_{n}=F_{c}-mg\cos\theta$$

**Figure 12:**
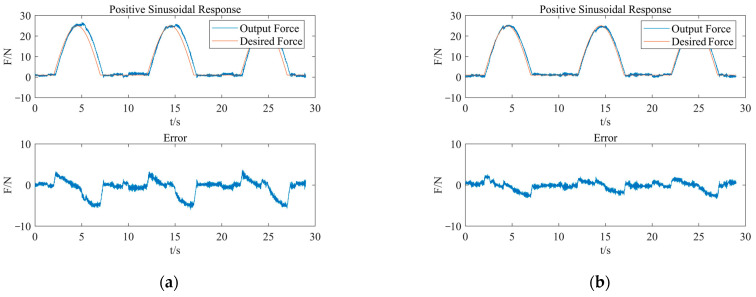

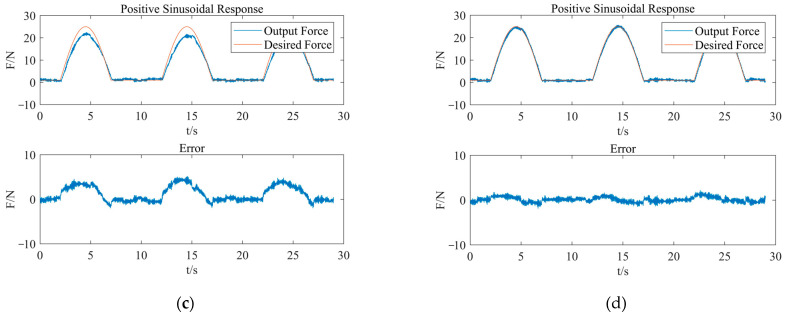
Forward sinusoidal tracking response analysis of contact force. (**a**) Open-loop control; (**b**) PID control; (**c**) fuzzy control; (**d**) fuzzy-RBF-PID control.

To maintain a constant contact force between the strengthening tool and the blade surface, the output force of the end-effector should be a trigonometric function related to *θ*. Therefore, the sinusoidal tracking experiment is required to test the tracking performance of the output force of the end-effector. The amplitude of the expected contact force is set to 25 N, and the frequency is set to 0.1 Hz. Considering that the actual output force signal is greater than 0 N, after checking the control performance of the control algorithm under the condition of continuous contact force, we took the portion of the expected force curve *F* ≥ 1 as the target curve. The test results are shown in Figure 12.

The control deviation of the system applying PID control is relatively small: in the range of −3 N~2.5 N. After the peak response force, the regulation delay lasts about 160 ms. PID control generally has high control accuracy, and the control deviation is mainly caused by a delay of output force due to system response hysteresis.

The feedback value of the output force in the fuzzy control system has an attenuation at the peak. Similarly to the step response, it results from a steady-state error generated by fuzzy control. In addition, the expected force curve and the response error curve both have sinusoidal variation. The system experiences an adjusting delay of approximately 60 ms after the peak.

Compared to the PID and fuzzy control methods, the fuzzy neural network PID control method has proven to be effective in adjusting the response deviation and delay of the output force. The system adjustment after the peak is delayed for about 45 ms, and the control deviation is kept within ±1.5 N. In terms of overall control performance, the fuzzy neural network PID control can significantly improve the adjusting speed of the PID control and offset the steady-state error in the fuzzy control. Consequently, the control accuracy, response speed, and tracking performance of the end-effector are effectively improved.

### 5.2. Experimental on the Output Force while Strengthening the Blade Surface

**Figure 13:**
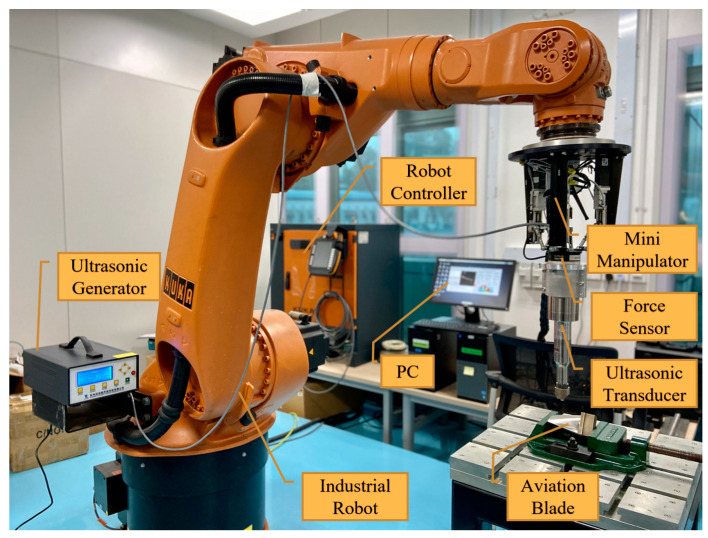
Experimental platform of robotic ultrasonic strengthening.

**Figure 14:**
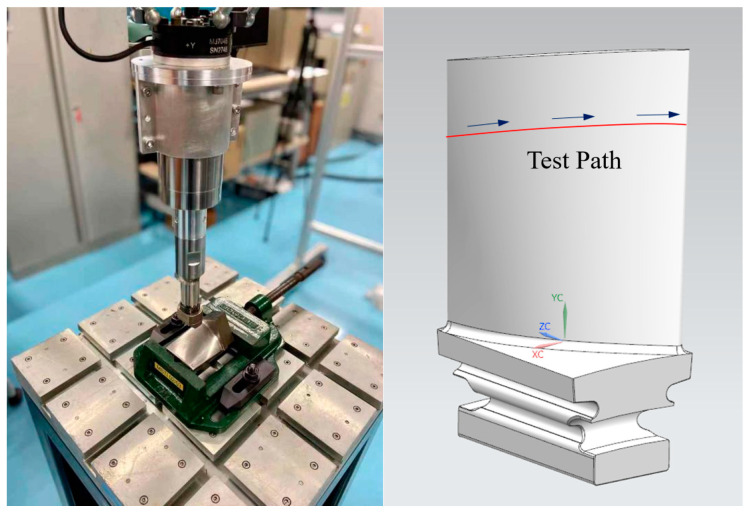
Contact force test on a single path.

A strengthening path on the blade was selected as the experimental trajectory, with the aim to study the performance of dynamically adjusting the output force of the compliance control algorithm, varying with the posture, in the process of ultrasonic strengthening. As shown in Figure 13, the experimental platform for the robotic ultrasonic strengthening of an aviation blade surface is built. The path of strengthening is planned based on the method proposed by previous research [38]. The blade workpiece is clamped on the workbench and positioned by the blade tenon, as shown in Figure 14. The target contact force is set to 25 N and, considering that the self-weight of the ultrasonic strengthening device is 1.73 kg, the actual expected output force of the end-effector can be obtained by Equation (21). In the experiment, the ultrasonic sinusoidal signal of the tool head is filtered by means of wavelet transformation, and the processed signal can be used as the actual contact force signal with working condition information in the process of robotic ultrasonic surface strengthening.

**Figure 15:**
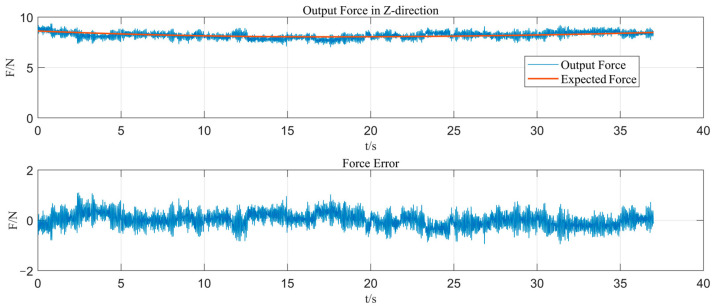
Response curve of output force on a single path with Fuzzy-RBF-PID control.

The experimental results are shown in Figure 15. The results indicate that the actual output force of the end-effector tracks the desired force very well; the output force error can be limited within ±1 N and satisfy the control accuracy requirement. The fuzzy RBF neural network is used to realize online self-tuning for the PID parameters and obtain the optimal parameter combination, so that the system can quickly approach the target value, improve the dynamic characteristics, and reduce the static error. The fuzzy neural network PID control not only has the advantage of the high steady-state accuracy of the PID control but also has the advantages of strong adaptability and flexible control of the fuzzy control. Therefore, the system can adjust the parameters self-adaptively in the dynamic pressure condition. The output force of the end-effector can accommodate the compliance control of contact force for the ultrasonic strengthening of an aviation blade surface due to good static and dynamic characteristics in the control process.

### 5.3. Surface Quality Evaluation for Robotic Ultrasonic Strengthening of Titanium Alloy Aviation Blade

**Figure 16:**
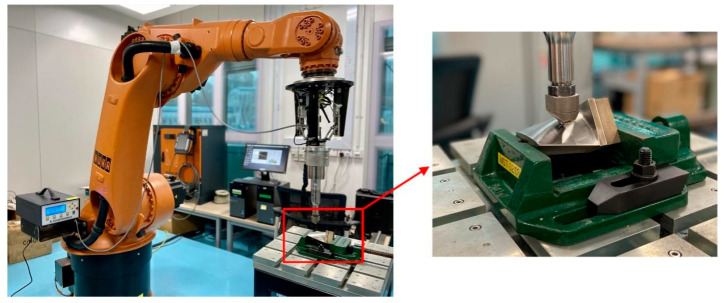
Robotic ultrasonic strengthening on aviation blade surface.

In order to verify the performance of the robotic compliance control for the ultrasonic strengthening of the aviation blade surface, the processing platform in Figure 13 is used for the regional strengthening of the titanium alloy aviation blade, as shown in Figure 16. The feed rate of robotic ultrasonic strengthening is set to 1.5 mm/s, and the process is repeated four times. The planned strengthening trajectory is input into the robotic controller. The target contact force signal of the end-effector is set to 25 N. The robotic ultrasonic surface strengthening is performed on the left half of the blade surface.

**Figure 17:**
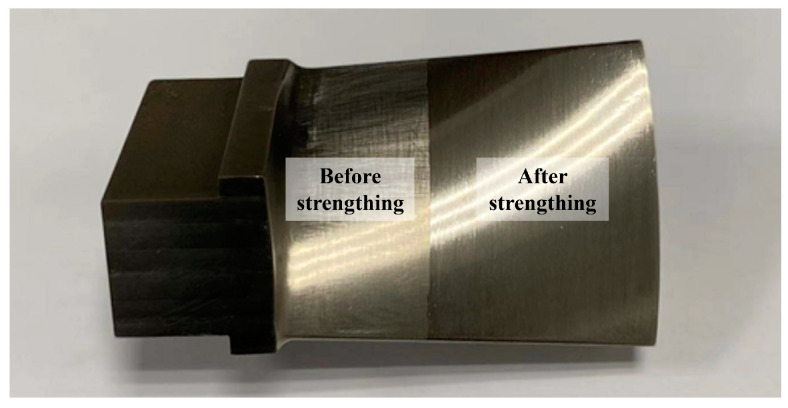
Aviation blade surface before and after ultrasonic surface strengthening.

The blade surface after robotic ultrasonic strengthening is shown in Figure 17. The quality of the blade surface is substantially improved, previous scratches and cutting textures have basically disappeared, and the surface becomes smooth and even.

**Figure 18:**
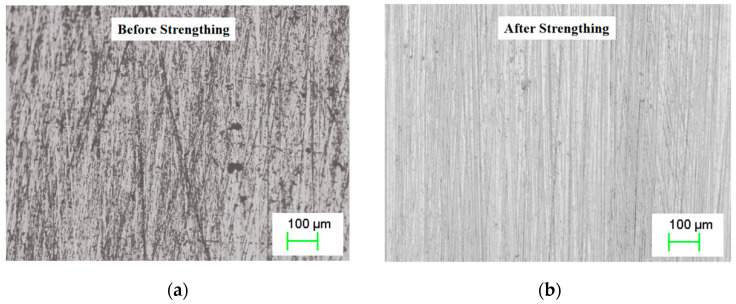
Microscopic morphology of the blade surface. (**a**) Before robotic ultrasonic strengthening; (**b**) after robotic ultrasonic strengthening.

Figure 18 shows the microscopic morphology of the blade surface. The figures show that regular and uniform strip-texture is formed on the surface of the titanium alloy blade after ultrasonic strengthening. There are no obvious over-strengthened or under-strengthened areas, which significantly improves the quality of the previously rough surface. After robotic ultrasonic strengthening, the surface roughness of the blade workpiece was reduced from Ra 2.7 μm to about Ra 0.8 μm, and the surface hardness was increased from 585 HL to about 672 HL. In future research, to make it easier to detect surface quality during the strengthening process, the advanced condition monitoring and fault diagnosis techniques for surfaces using machine vision systems can be applied [39,40].

The above figure shows that by applying the studied compliant force control strategy, the contact force output by the end-effector can be kept constant during the machining process to achieve a uniform and high-quality strengthening surface. It is also proven that, for the surface strengthening of a titanium alloy aviation blade, the application of industrial robot technology can achieve good and stable strengthening quality, thereby providing a new technical solution for the surface strengthening of aviation blades.

## 6. Conclusions

This paper proposes a robotic compliance control strategy for the ultrasonic strengthening of an aviation blade surface. The output force of the end-effector is regulated using an intelligent control method to achieve compliant contact between the ultrasonic strengthening tool and the blade surface under multi-impact and vibration conditions. The key contributions and conclusions are summarized as follows:

(1) The compliance control algorithm can improve the response performance of the system. The control algorithm utilizes the Fuzzy-RBF-PID method to control the contact force output of the end-effector, providing self-adaptability for the PID controller by utilizing the mapping relationship between the input and output of the fuzzy RBF neural network. Compared to conventional control methods, the proposed approach can enhance the system’s adjusting speed (with a max-delay of 45 ms), reduce overshoot, and significantly improve the regulation performance of the end-effector, as well as track the robustness of the dynamic system.

(2) The compliance control algorithm can realize accurate tuning for output force during blade strengthening. The experimental results of the output force control algorithm indicate that the fuzzy neural network PID control can effectively improve the response performance of the output force and tuning accuracy of the end-effector. The steady-state error problem in the process of fuzzy control can also be eliminated effectively. Through experimental verification, the dynamic tracking error of the output force can be kept within ±1 N.

(3) An experimental platform for the robotic ultrasonic strengthening of the aviation blade surface is built to verify the effectiveness and feasibility of the proposed compliance control strategy. After robotic ultrasonic strengthening, the regular and uniform strip-texture is formed on the surface of the titanium alloy blade, and a high-quality strengthening surface is obtained. The surface roughness is reduced by about 70% from Ra 2.7 μm to Ra 0.8 μm, and the surface hardness increases by about 15% from 585 HL to 672 HL. Therefore, it provides a feasible reference for the practical application of robotic compliance control technology for the ultrasonic strengthening of an aviation blade surface.

## Data Availability

The data of this research are being used for further extended research and can be made available in due course.
